# A novel case of concurrent occurrence of demyelinating-polyneuropathy-causing *PMP22* duplication and *SOX10* gene mutation producing severe hypertrophic neuropathy

**DOI:** 10.1186/s12883-021-02256-y

**Published:** 2021-06-25

**Authors:** Nozomu Matsuda, Koushi Ootsuki, Shunsuke Kobayashi, Ayaka Nemoto, Hitoshi Kubo, Shin-ichi Usami, Kazuaki Kanani

**Affiliations:** 1 Department of Neurology, Fukushima Medical University, 1 Hikarigaoka, Fukushima, Fukushima 960-1295 Japan; 2 Department of Otorhinolaryngology, Fukushima Medical University, 1 Hikarigaoka, Fukushima, Fukushima 960-1295 Japan; 3 Department of Neurology, Teikyo University School of Medicine, 2-11-1 Kaga, Itabashi, Tokyo, 173-8606 Japan; 4 Advanced Clinical Research Center, Fukushima Medical University, 1 Hikarigaoka, Fukushima, Fukushima 960-1295 Japan; 5 Preparing Section for New Faculty of Medical Science, Fukushima Medical University, 1 Hikarigaoka, Fukushima, Fukushima 960-1295 Japan; 6 Department of Hearing Implant Sciences, Shinshu University School of Medicine, 3-1-1 Asahi, Matsumoto, Nagano, 390-8621 Japan

**Keywords:** Charcot–Marie–tooth disease, Waardenburg syndrome, PMP22, SOX10, Hypertrophic neuropathy, Whole-body MRI, MR neurography

## Abstract

**Background:**

Hereditary motor and sensory neuropathy, also referred to as Charcot–Marie–Tooth disease (CMT), is most often caused by a duplication of the peripheral myelin protein 22 (*PMP22*) gene. This duplication causes CMT type 1A (CMT1A). CMT1A rarely occurs in combination with other hereditary neuromuscular disorders. However, such rare genetic coincidences produce a severe phenotype and have been reported in terms of “double trouble” overlapping syndrome. Waardenburg syndrome (WS) is the most common form of a hereditary syndromic deafness. It is primarily characterized by pigmentation anomalies and classified into four major phenotypes. A mutation in the SRY sex determining region Y-box 10 (*SOX10*) gene causes WS type 2 or 4 and peripheral demyelinating neuropathy, central dysmyelinating leukodystrophy, WS, and Hirschsprung disease. We describe a 11-year-old boy with extreme hypertrophic neuropathy because of a combination of CMT1A and WS type 2. This is the first published case on the co-occurrence of CMT1A and WS type 2.

**Case presentation:**

The 11-year-old boy presented with motor developmental delay and a deterioration in unstable walking at 6 years of age. In addition, he had congenital hearing loss and heterochromia iridis. The neurological examination revealed weakness in the distal limbs with pes cavus. He was diagnosed with CMT1A by the fluorescence in situ hybridization method. His paternal pedigree had a history of CMT1A. However, no family member had congenital hearing loss. His clinical manifestation was apparently severe than those of his relatives with CMT1A. In addition, a whole-body magnetic resonance neurography revealed an extreme enlargement of his systemic cranial and spinal nerves. Subsequently, a genetic analysis revealed a heterozygous frameshift mutation c.876delT (p.F292Lfs*19) in the *SOX10* gene. He was eventually diagnosed with WS type 2.

**Conclusions:**

We described a patient with a genetically confirmed overlapping diagnoses of CMT1A and WS type 2. The double trouble with the genes created a significant impact on the peripheral nerves system. Severe phenotype in the proband can be attributed to the cumulative effect of mutations in both *PMP22* and *SOX10* genes, responsible for demyelinating neuropathy.

## Background

Hereditary motor and sensory neuropathy is also known as Charcot–Marie–Tooth disease (CMT). It results from mutations in a variety of genes [1, 2]. The majority of CMT cases are caused by mutations in the peripheral myelin protein 22 (*PMP22*) gene [3]. PMP22 is primarily responsible for myelin adhesion and maintenance. A duplication in the *PMP22* gene produces CMT type 1A (CMT1A). In contrast, a deletion in this gene produces hereditary neuropathy with liability to pressure palsy (HNPP) [4]. Patients with CMT1A display a reduced velocity of nerve conduction and the usual age of clinical onset is in the first decade of life. Herein, we describe a family in which the proband presented with atypical CMT1A at the age of 11 years. His father and sister had been diagnosed with typical CMT1A. However, his clinical manifestations and electrophysiological findings were more severe than those of the family members. A whole-body magnetic resonance neurography (WB-MRN) revealed an extreme enlargement of the cranial and spinal nerves. The proband possessed a duplication of the *PMP22* gene, consistent with CMT1A. Moreover, we identified a mutation c.876delT (p.F292Lfs*19) in the SRY sex determining region Y-box 10 (*SOX10*) gene. This mutation was associated with Waardenburg syndrome (WS) type 2 or 4 and peripheral demyelinating neuropathy, central dysmyelinating leukodystrophy, WS, and Hirschsprung disease (PCWH) [5–9]. The severe neuropathy of the proband was most likely because of the double trouble with *PMP22* and *SOX10* genes. The aforementioned genes play important roles in developing and maintaining the myelin sheath in the peripheral nervous system. We believe that this case makes a significant contribution to the literature because it is the first published case on the concomitant occurrence of CMT1A and WS type 2.

## Case presentation

**Fig. 1:**
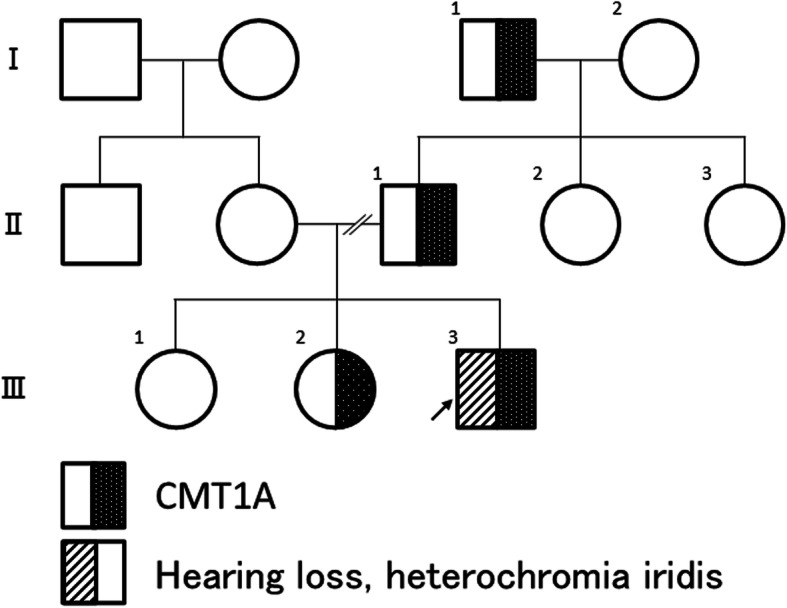
Pedigree chart. The proband, sister, and some of his paternal family members have similar symptoms of polyneuropathy with foot deformities. Only the proband has been diagnosed with congenital hearing loss and heterochromia iridis

The proband (III:3) was diagnosed with congenital hearing loss on a newborn hearing screening test. Furthermore, he manifested delayed motor developmental milestones. He presented with unstable walking at 6 years of age. He did not face any problem with scholastic attainments in elementary school. Nonetheless, he was not good at physical education. He consulted our otolaryngologist at the age of 10 years for hearing loss. He was referred to our department in 2015 for gait disturbance at 11 years of age. He had a history of intestinal obstruction at the age of 2 years. However, the symptoms improved with conservative treatment and did not require surgery. His family history included gait disturbance with pes cavus in his paternal pedigree. There was no family history of congenital hearing loss (Fig. 1). He presented with heterochromia iridis during the physical examination. Moreover, there were no partial hypopigmentation of the skin and hair, dystopia canthorum, upper limb abnormalities, and Hirschsprung disease. There were no cafe-au-lait spots on his skin. He presented with bilateral sensorineural hearing loss during the neurologic examination. However, there was no abnormality in higher cognitive functions. Furthermore, his pupil, eye movement, facial sensation, facial muscles, swallowing, and tongue were intact. He had pes cavus, and mild weakness of the distal limb muscles. The quantified muscle strength on the Medical Research Council (MRC) scale (right, left) was (4, 4) in the abductor pollicis brevis, abductor digiti minimi, tibialis anterior, and gastrocnemius muscles, respectively. The muscle volumes were mostly preserved except for mild atrophy in the intrinsic hand and foot muscles. In addition, he showed a wide-based ataxic gait. The finger-nose and knee-heel test showed normal results. The patient did not complain of numbness. However, there was a profound decrease in his vibration and position senses with normal pinprick and cold senses. He displayed a positive Romberg sign. While the deep tendon reflexes were diminished, the bilateral plantar responses were flexor. On assessing his right median motor nerve conduction, we found a prolonged distal latency (12.1 ms) and a profound conduction slowing (9.1 m/s). Moreover, he displayed reduced compound muscle action potential amplitudes (1.4 mV) and severe demyelinating neuropathy.

The fluorescence in situ hybridization method showed a duplication encompassing the *PMP22* gene on chromosome 17p11.2–12, thus confirming CMT1A. After obtaining written informed consent from his mother, we obtained a genomic DNA sample from the leukocytes of the proband and his mother. Subsequently, we used a next-generation gene sequencer to analyze mutations in the WS-associated genes; the detailed information is described elsewhere [10]. The patient carried a heterozygous frameshift mutation c.876delT (p.F292Lfs*19) in the *SOX10* gene and was diagnosed with WS type 2. His mother did not have this mutation.

**Fig. 2:**
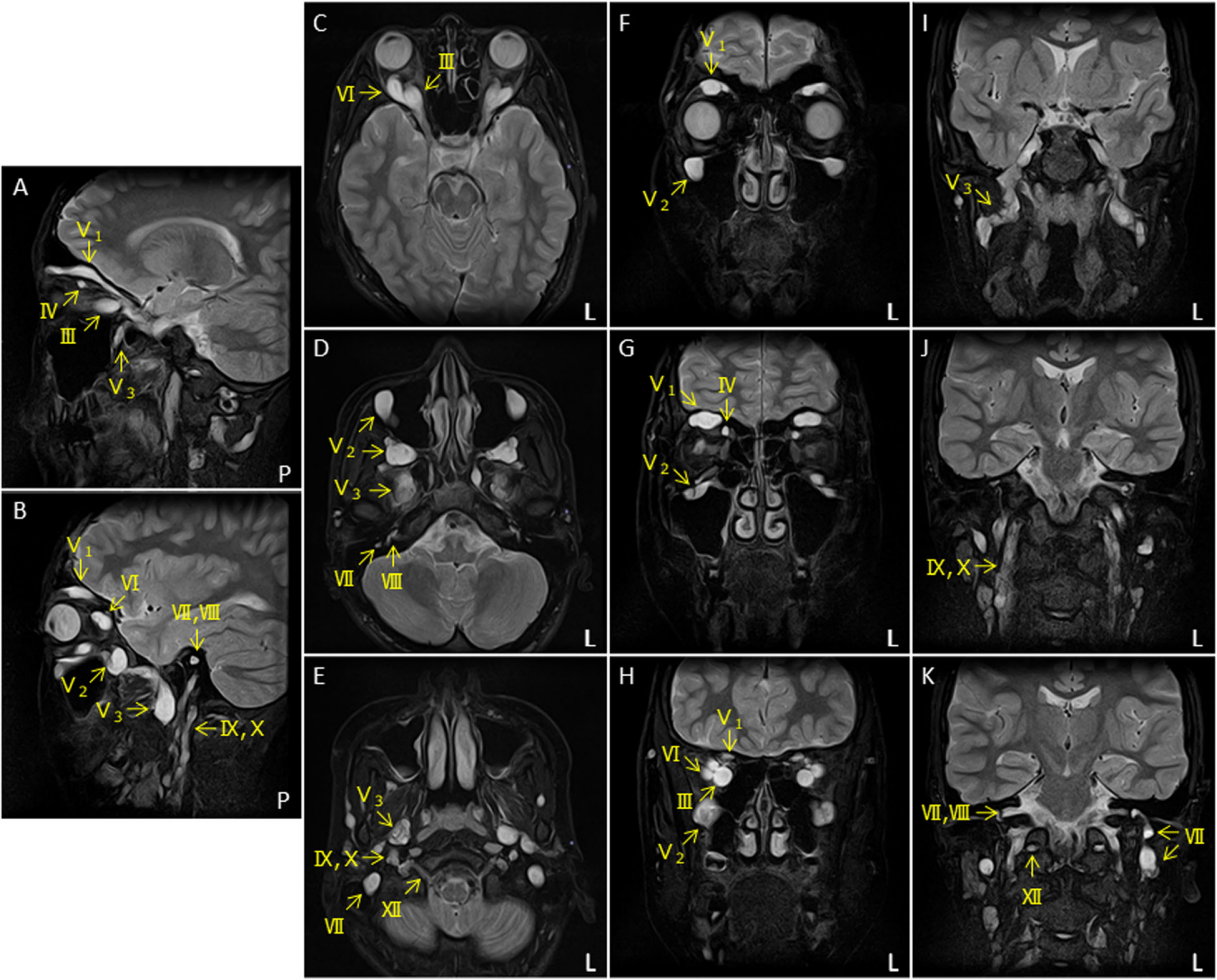
Magnetic resonance imaging of the cranial nerves. Sagittal (**A**-**B**), axial (**C**-**E**), and coronal (**F**-**K**) T2-weighted images reveal thickened cranial nerves except the olfactory and optic nerves. Abbreviations, III: oculomotor nerve, IV: trochlear nerve, V1: ophthalmic nerve, V2: maxillary nerve, V3: mandibular nerve, VI: abducens nerve, VII: facial nerve, VII: auditory nerve, IX: glossopharyngeal nerve, X: vagus nerve, XII: hypoglossal nerve, L: left, and P: Posterior

**Fig. 3:**
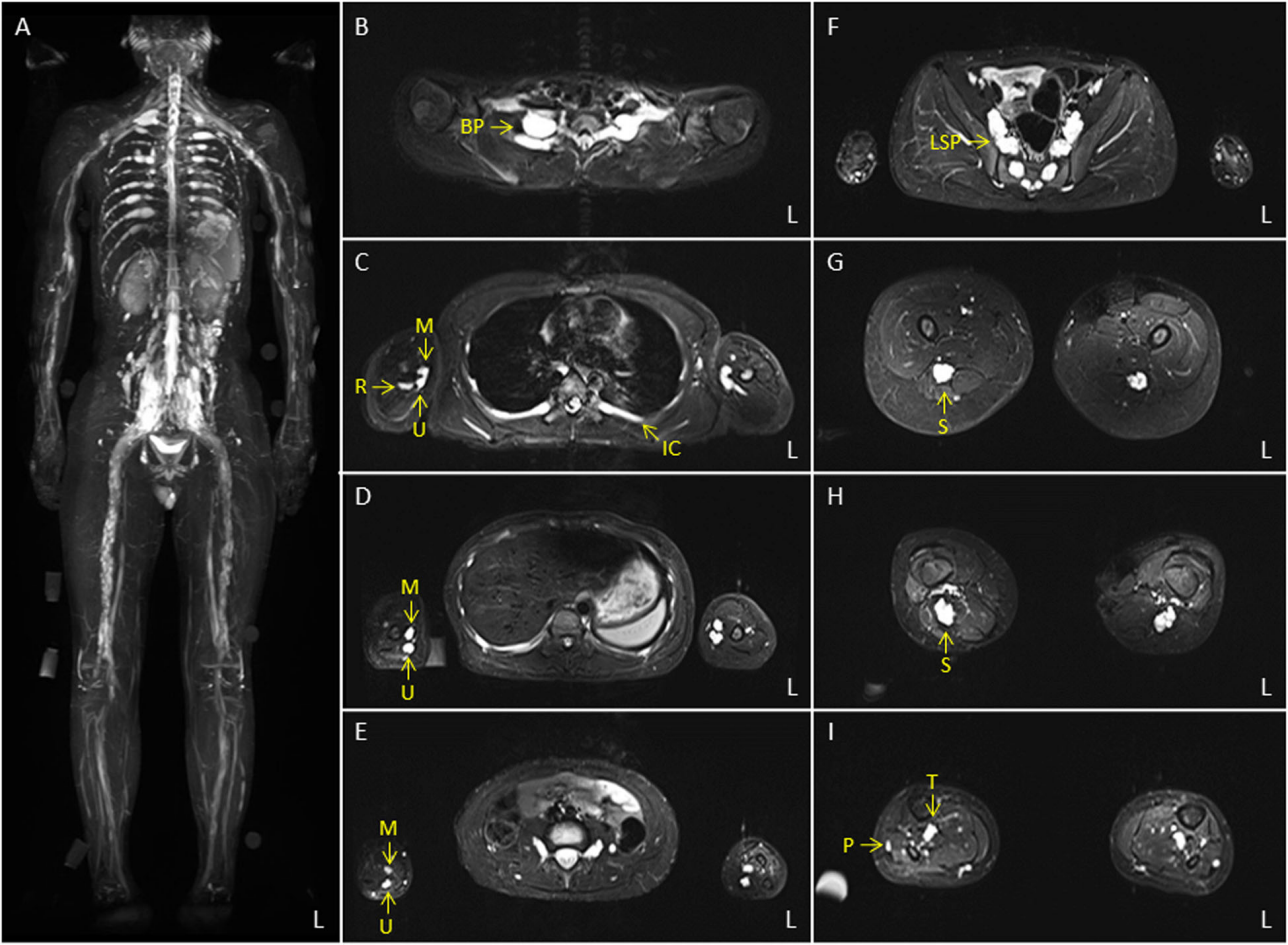
Whole-body magnetic resonance neurography (WB-MRN) with short tau inversion recovery (STIR) sequences. WB-MRN by maximum intensity projection (**A**) and axial STIR images (**B**-**I**) demonstrate an extreme enlargement of the entire peripheral nervous system with some nodular changes. Abbreviations, BP: brachial plexus, IC: intercostal nerve, L: left, LSP: lumbosacral plexus, M: median nerve, P: peroneal nerve, R: radial nerve, S: sciatic nerve, and U: ulnar nerve

A cranial MRI demonstrated extreme hypertrophic changes in the cranial nerves except in the olfactory and optic nerves. We observed severe and diffuse enlargements in the oculomotor (III), trochlear (IV), ophthalmic, maxillary and mandibular branches of the trigeminal (V1, V2, V3), abducens (VI), facial (VII), auditory (VII), glossopharyngeal (IX), vagus (X), and hypoglossal (XII) nerves (Fig. 2). Moreover, there were no white matter lesions as observed in cerebral leukodystrophy. A WB-MRN by a projection image of maximum intensity on a short tau inversion recovery (STIR) sequence also revealed severe hypertrophic changes of the systemic peripheral nervous system. In other words, we observed hypertrophic changes in the spinal nerves that innervate the limbs and trunk from the nerve roots. Moreover, an axial STIR image revealed nodular changes on some parts of the thickened nerves (Fig. 3).

**Fig. 4:**
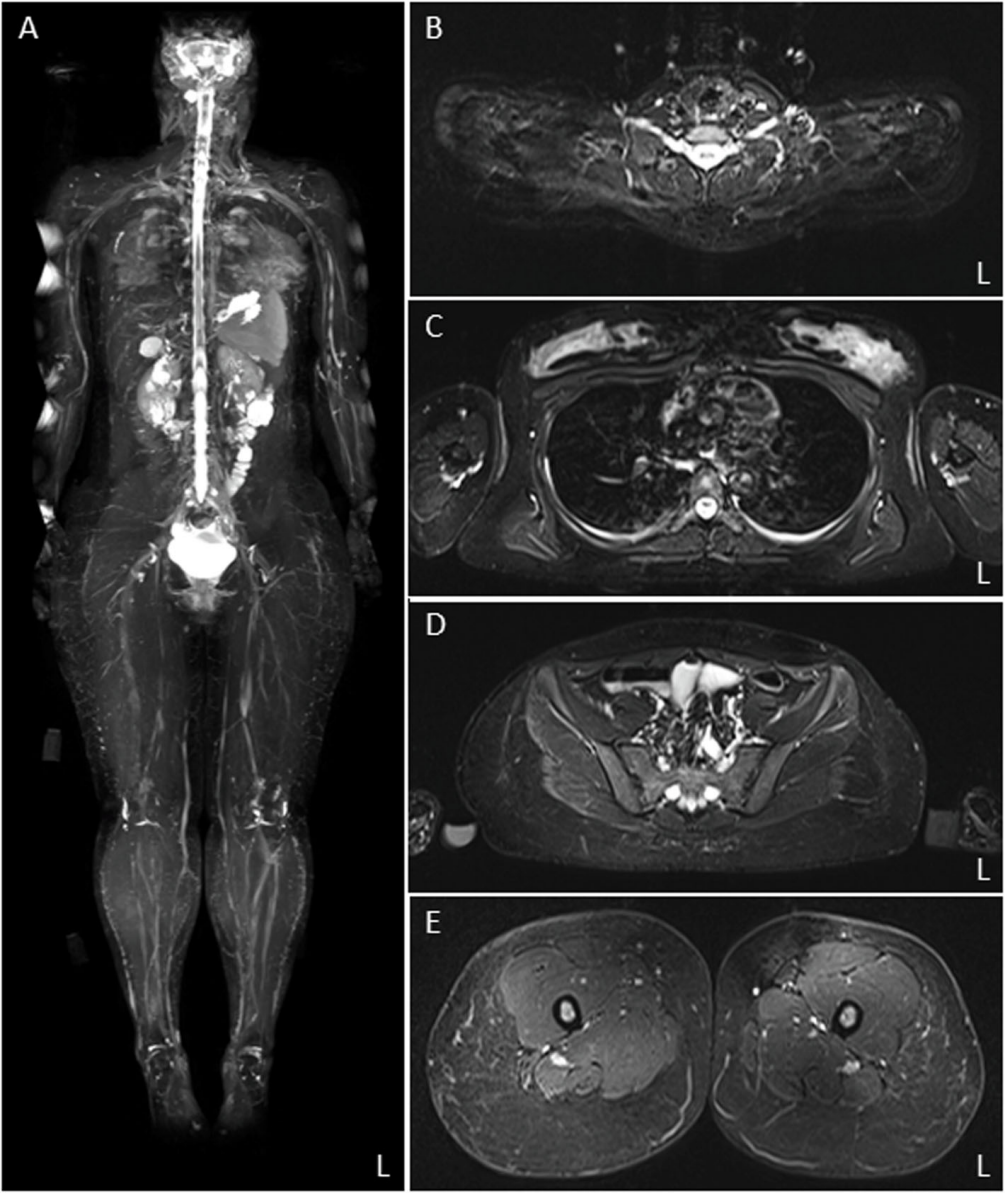
Magnetic resonance imaging with short tau inversion recovery sequences in the proband’s sister with CMT1A. Coronal magnetic resonance neurography (**A**), and axial images (**B**-**E**) do not reveal an extreme nerve thickening, as observed in the proband

We examined the 19-year-old sister (III:2) of the proband. She had pes cavus and displayed slight weakness of the tibialis anterior muscle on using the MRC scale (5-, 5-). While she revealed a decreased vibration sense, deep tendon reflexes were absent. However, her gait was normal. Right motor nerve conduction studies revealed a prolonged distal latency (9 ms in the median, 7.3 ms in the ulnar nerve), conduction slowing (18.6 m/s in the median, 17.8 m/s in the ulnar nerves), and relatively preserved amplitude (5 mV in the median, 6.3 mV in the ulnar nerve). Therefore, nerve conduction studies displayed uniform conduction slowing because of CMT1A. However, WB-MRN did not show any extreme hypertrophic changes in the peripheral nerves (Fig. 4).

## Discussion and conclusions

Herein, we described a 11-year-old boy with hypertrophic demyelinating neuropathy caused by the duplication and a heterozygous frameshift mutation in the *PMP22* (CMT1A) and *SOX10* genes (WS type 2), respectively. The aforementioned genes are responsible for developing and maintaining the myelin sheath in peripheral nerves [11, 12]. This is the first published case on the co-occurrence of CMT1A and WS type 2.

His paternal family and sister had CMT1A. However, his clinical presentation and nerve conduction study revealed a greater disease severity, compared to his family member with CMT1A. Unlike the family members, the proband presented with congenital hearing loss and heterochromia iridis. Thus, he was diagnosed with WS type 2. WS is the most common form of autosomal dominant congenital hearing loss. Moreover, its incidence is speculated to be 1/42000 [5]. It is characterized by sensorineural hearing loss and pigmentation abnormalities, such as heterochromia and depigmented spots on the skin and hair. Researchers have classified WS into four types. While WS type 1 has a dystopia canthorum, type 2 does not have this anomaly. WS type 3 is characterized by an upper limb abnormality, in addition to dystopia canthorum. In contrast, a patient with WS type 4, also known as Waardenburg-Shah syndrome suffers from Hirschsprung’s disease. WS is genetically heterogenous and mutations in the *SOX10* gene are partially responsible for WS type 2 and 4 [13]. In addition, this mutation produces severe cerebral leukodystrophy and peripheral demyelinating neuropathy, in conjunction with a WS type 4 phenotype, also known as PCWH [7–9].

WB-MRN revealed severe hypertrophy of the systemic cranial and spinal nerves in the proband. Previous studies using ultrasound and MRI in patients with CMT1A have reported uniform spinal nerve enlargements [14, 15]. Yiu and colleagues conducted a sonographic study and reported a substantial increase in the cross-sectional areas of the median, ulnar, tibial, and sural nerves (1.9- to 3.5- fold increase) in children with CMT1A (mean age: 11.4 years), compared to healthy controls [14]. Furthermore, Shibuya and colleagues conducted MRN with a 3-D reconstruction of STIR images. They reported on hypertrophic changes from the cervical nerve root to the brachial plexus in patients with CMT1A [15]. However, the proband had a significantly greater enlargement of the spinal nerves than did his sister and the patients reported in previous studies. Furthermore, he displayed an enlargement of the cranial nerves. This characteristic is rarely reported in CMT [16, 17].

According to several studies, a patient having two separate mutations in neuromuscular disease-associated genes might develop an unusual severe phenotype. Such a condition is sometimes referred to as a “double trouble” overlapping syndrome. Previous studies have reported genetically confirmed cases, including a combination of CMT1A/CMTX (gap junction β-1: *GJB1* gene) [18], CMT1A/CMT1C (lipopolysaccharide-induced tumor necrosis factor-α factor: *LITAF* gene) [19], CMT1A/myotonic dystrophy (dystrophia myotonica protein kinase: *DMPK* gene) [18], CMT1A/facioscapulohumeral muscular dystrophy [20], and HNPP/adrenomyeloneuropathy (ATP binding cassette subfamily D member 1: *ABCD1* gene) [18]. The aforementioned concomitant mutations might exert a cumulative effect and produce a novel and severe phenotype. Hence, severe hypertrophic changes of the peripheral nerves in the proband can be attributed to the cumulative effect of mutations in both *PMP22* and *SOX10* genes, responsible for demyelinating neuropathy.

**Fig. 5:**
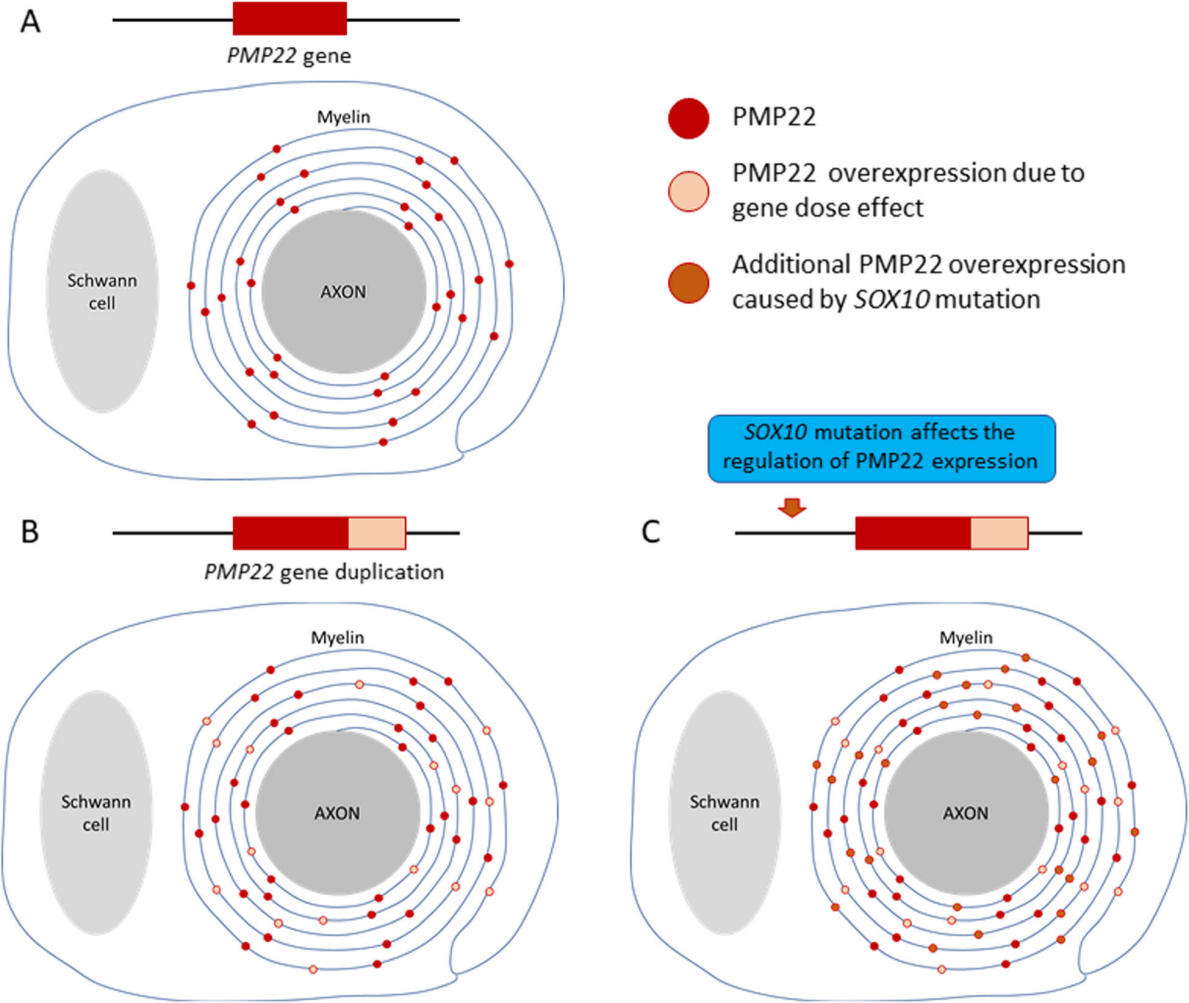
A hypothetical pathophysiology of extreme hypertrophic neuropathy because of *PMP22* and *SOX10* gene mutations. The patient with CMT1A (**B**) has an additional copy of the *PMP22* gene. The PMP 22 proteins are overexpressed in the Schwan cell and myelin layers, compared to healthy controls (**A**). Aberrant interaction between the *SOX10* and *PMP22* genes may cause an additional overexpression of the PMP22 protein in the proband (**C**)

Furthermore, we hypothesized that an aberrant interaction of the *SOX10* gene mutation and *PMP22* gene duplication is likely to cause an overexpression of PMP22 in the myelin sheath. Copy number variants (CNVs) have been recently found responsible for numerous inherited disorders [21, 22]. CNVs are defined as chromosomal structural variants with a gain or loss of genetic sequences by at least 50 bp. CMT1A is one of the presentative genomic disorders associated with CNVs. Furthermore, it is characterized by a gain of a 1.4 mb segment at the 17p12 locus and harbors an additional copy of the *PMP22* gene. In contrast, HNPP occurs because of a deletion of the chromosome and is characterized by the loss of a 1.4 mb segment. Several researchers have reported on the triplication of the 17p12 locus in some patients with severe CMT1A phenotype [23]. A quantitative immunohistochemical study using sural nerve biopsies reported on PMP22 overexpression in patients with CMT1A; however, it was reduced in those with HNPP [24, 25]. Therefore, the dosage of the *PMP22* gene is the most likely pathogenic mechanism that explains the severity of CMT1A. SOX10 is a transcription factor encoded on the long arm of chromosome 22. It plays an important role in the development of the central and peripheral nervous systems, enteric cells, and melanocytes [11, 26]. The *SOX10* gene is expressed at all developmental stages of Schwann cells and is required for the differentiation of embryonic and mature Schwann cells. Furthermore, SOX10 upregulates the expression of the early growth response protein 2 (Egr2) before myelination in Schwann cells. Egr2 is required for the formation and maintenance of the myelin sheath. In addition, it activates several myelin-related genes. SOX10 plays synergistically with Egr2 to regulate the expression of PMP22 and other myelin-related genes, such as myelin protein zero [27, 28]. However, the expression of *PMP22* gene is temporally regulated to control the critical level of PMP22 for an appropriate myelin formation. Therefore, we speculated that an aberrant interaction between the *SOX10* and *PMP22* genes caused an overexpression of the PMP22 protein in the peripheral nervous system of the proband, thus resulting in severe hypertrophic neuropathy [29] (Fig. 5).

This case report was subject to several limitations. First, only the proband had symptoms of WS, which indicates that he has a de novo mutation, but we were unable to conduct a genetic screening test for *SOX10* in his family member with CMT1A. Second, we did not perform a nerve biopsy and could not directly confirm the patient’s peripheral nerve pathology, especially the increased expression of the PMP22 protein. Finally, to date the precise mechanism of the influence PMP22 expression by translation factors such as SOX10 is not clear. Further basic research is required to establish our hypothesis.

## Data Availability

All data supporting our findings are provided within the manuscript.

## Abbreviations

CMT: Charcot–Marie–Tooth disease
CNVs: Copy number variants
Egr2: Early growth response protein 2
HNPP: Hereditary neuropathy with liability to pressure palsy
MRC: Medical Research Council
PCWH: Peripheral demyelinating neuropathy, central dysmyelinating leukodystrophy, Waardenburg syndrome and Hirschsprung disease
PMP22: Peripheral myelin protein 22
SOX10: SRY sex determining region Y-box 10
STIR: Short tau inversion recovery
WS: Waardenburg syndrome
WB-MRN: Whole-body magnetic resonance neurography
